# The Role of LTB4 in Obesity-Induced Insulin Resistance Development: An Overview

**DOI:** 10.3389/fendo.2022.848006

**Published:** 2022-03-22

**Authors:** Irineu Otavio Marchiori Callegari, Alexandre Gabarra Oliveira

**Affiliations:** Department of Physical Education, São Paulo State University (UNESP), Rio Claro, Brazil

**Keywords:** inflammation, insulin resistance, polyunsaturated fatty acids (PUFA), adaptive immune system, obesity

## Introduction

Obesity prevalence in a global context has been identified as a trigger that causes increased risk for developing chronic diseases, largely due to the presence of subclinical inflammation (1). In fact, changes in glucose and fatty-acid metabolism have been associated with diets involving excessive consumption of sucrose and saturated fat (2). This dietary pattern leads to changes in immune system activity such as an increase in macrophage infiltration and its polarization towards the M1 phenotype, which in turn deregulates the M1/M2 ratio, reducing the degree of tissue remodeling, and local homeostasis of insulin-sensitive tissues. In addition, there is a relationship between the M1/M2 imbalance and proinflammatory markers of adaptive immunity in the context of chronic diseases (3). In this regard, the role played by leukotriene B4 (LTB4), a proinflammatory lipid mediator produced from arachidonic acid (AA), has been highlighted (4). In this context, the high-fat diet (HFD) is probably a strong stimulus for LTB4 synthesis as this diet increases AA levels and consequently the production of lipid mediators in the visceral adipose tissue (5). In other words, a potent stimulus for LTB4 synthesis is possibly HFD-induced lipotoxicity. The LTB4 synthesis is enhanced in other instances than obesity since the LTB4/LTB4R1 axis is important for the immune system during an acute infection (6). In addition, LTB4 is also increased in atherosclerosis and arthritis, pathologies that are associated with chronic inflammation (7–9). Despite the increase in LTB4/LTBR1 being not only specific for obesity, it is well documented that LTB4 has a pivotal role in sustaining proinflammatory status in the context of obesity and insulin resistance (IR), due to its capacity to promote migration of the M1 macrophage when coupled to its receptor, referred to as LTB4R1 (10). Furthermore, Li and colleagues (11) have established the relationship between the LTB4-LTB4R1 system and glucose metabolism in *in vivo* and *in vitro* studies. Indeed, they have shown that LTB4 treatment impairs insulin-stimulated glucose transport by decreasing insulin-stimulated Akt phosphorylation due to IRS-1 serine phosphorylation, which in turn inhibits Glut4 translocation in L6 myocytes. In contrast, they also observed that the LTB4R1 inhibitor (CP105696) restores insulin sensitivity, as evidenced by increasing the glucose infusion rate during hyperinsulinemic-euglycemic clamp studies of C57BL rodents fed on a HFD for 14 weeks. Given the relevance obtained in proving the role played by LTB4 in the context of obesity and IR, in the current article we focus on evidence that shows LTB4 acting on inflammation developed due to HFD-induced obesity, along with the potential strategies used to mitigate the connection between LTB4-LTB4R1.

## The Underlying Mechanisms of Activation of the LTB4-LTB4R1 Axis in Insulin Resistance

**Figure 1:**
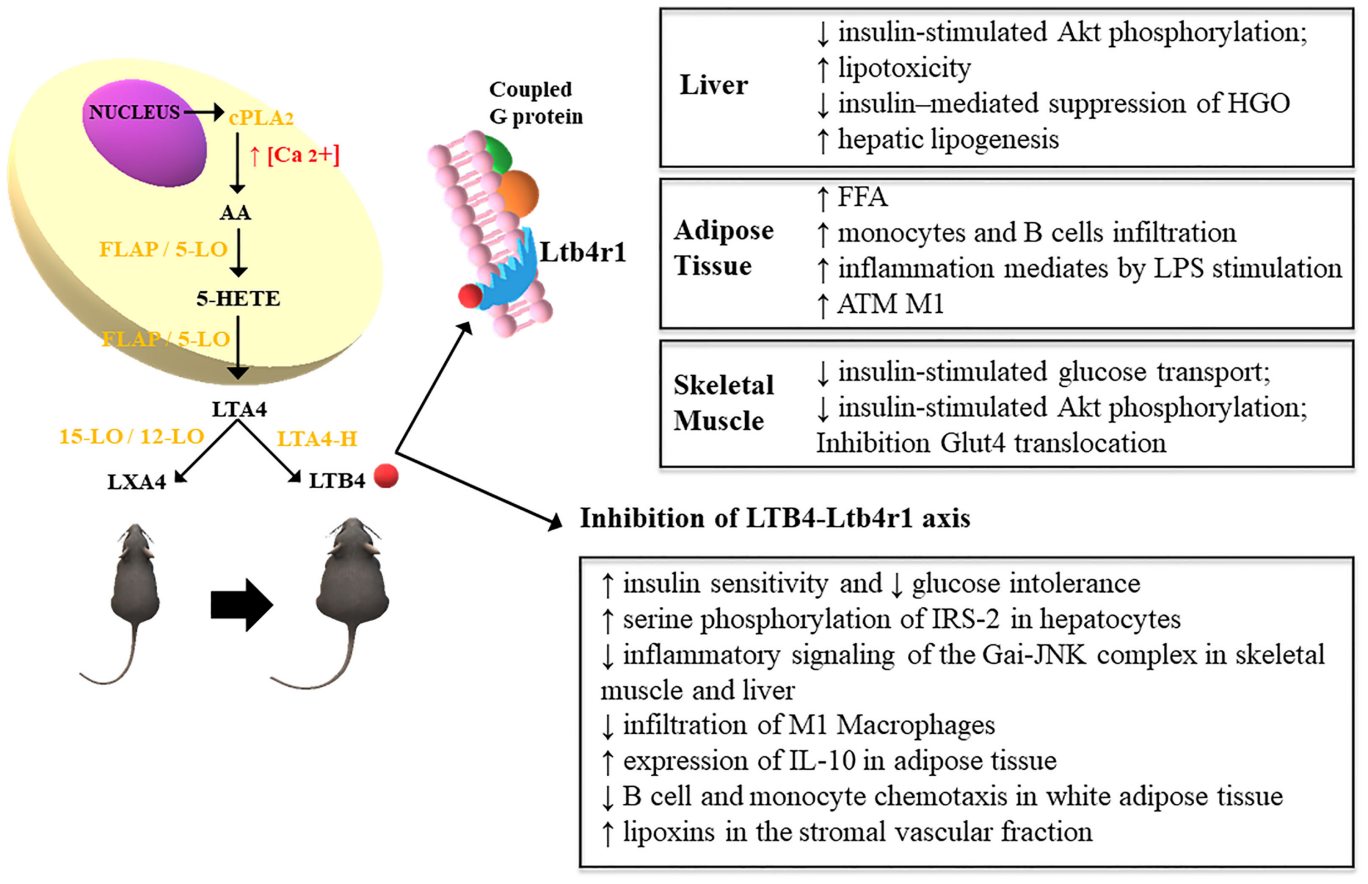
Leukotriene synthesis occurs from the release of arachidonic acid and the action of cytosolic phospholipase A2 (cPLA2) due to the increase in intracellular calcium, which increases the expression of 5-lipoxygenase (5-LO), which translocates to the cell nucleus by interacting with 5-LO membrane anchor activating protein (FLAP). This leads to the formation of 5-hydroperoxyeicosatetraenoic acid (5-HETE) which gives rise to the formation of LTA4, converted to LTB4 by the action of the enzyme LTA4 hydrolase (LTA4-H) in obese rodents. In turn, lean animals convert LTA4 into lipoxins (LXA4) through the action of the enzyme 15/12-LO (12). The increased expression of LTB4 exerts inflammatory effects through the high-affinity interaction with its G protein-coupled receptor, Ltb4r1. This mechanism impairs the metabolism of glucose and fatty acids in the liver, TA and skeletal muscle. These effects are attenuated through the pharmacological inhibition of LTB4R1 (11).

When Li and colleagues (11) achieved the inhibition of the LTB4/LTB4R1 axis, either by knock out or pharmacological inhibition of LTB4R1, and observed that LTB4 can directly promote IR, the understanding of the underlying mechanisms became intriguing. In this regard, they uncovered that the G protein-coupled receptor (Gαi) and c-Jun N-terminal kinases (JNK) activity as the mediators of LTB4/LTB4R1 deleterious effects in obesity. To elucidate the role of Gαi the authors pre-treated myocytes with the Gαi pharmacological inhibitor, pertussis toxin, that resulted in blockade of LTB4 effects to impair insulin signaling (**Figure 1**). In contrast, as LTB4-LTB4R1 can induce 307 serine phosphorylation of IRS-1 in obesity, which in turn interferes in the insulin signaling pathway, the same authors also assessed the effects of the inhibition of serine kinases IKK, ERK and JNK. While knockdown of IKK and ERK had no effects on LTB4-induced IR, the pharmacological inhibition of JNK blunted the negative effects of LTB4 in glucose transport in L6 myocytes. Taken together, the results obtained in the study by Li and colleagues indicate that LTB4 can cause IR by a mechanism involving JNK activity.

Interestingly, the relationship among LTB4, LTB4R1 and Gαi had already been addressed before by Wang and co-workers (13), who demonstrated that this axis could modulate microRNAs associated with activation of the MyD88-mediated macrophage. Indeed, macrophages harvested from the peritoneum of mice and cultured 24 h with LTB4 showed an increase in the levels of miR-155, a micro-RNA able to reduce SOCS1 expression, which in turn increases MyD88 levels allowing higher toll-like receptor (TLR) activation. They also demonstrated that LTB4-induced miR-155 expression is mediated by AP-1 since the pretreatment with AP-1 inhibitor SR11302 blunted the effects of LTB4 on miR-155 expression. In accordance, Gaudreault and coworkers (14) previously demonstrated that the LTB4-LTB4R1 axis can enhance TLR-induced AP-1 activity through TAK1 phosphorylation. Furthermore, LTB4 treatment alone is able to induce proinflammatory effects, such as the release of RANTES and IL-6 (15). Taken together, these studies highlighted that one of mechanisms by which the LTB4-LTB4R1 axis induces inflammation is through enhancing the TLR signaling pathway.

## Is There a Correlation Between LTB4-LTBR1 and Polyunsaturated Fatty Acids?

Western diets usually have a huge level of omega (ω)-6 compared to a content of ω-3; this difference achieves a ratio of 20:1 instead of 1:1 which is the recommendation for health, thus increasing risk of some diseases (16). Such imbalance might result in increased inflammation, since the products derived from ω-6 such as prostaglandin E2 (PGE2) and LTB4 are strong inflammatory mediators compared to similar mediators that come from ω-3 (17). In this context, Li J. and colleagues (17) investigated the importance of ω-3 and ω-6 polyunsaturated fatty acids (PUFA) in the development of obesity and IR. For this purpose, they utilized Fat-1 transgenic mice that convert ω-6 into ω-3, i.e., these animals not only showed high levels of ω-3 but also reduction of ω-6. These alterations in the omega content increased the energy expenditure that confers to these animals a resistance to develop obesity during an HFD challenge. Interestingly, fat-1 mice exhibited increased glucose tolerance and even insulin sensitivity, i.e., these animals showed a protection to HFD-induced IR. The results also showed that compared to wild type, the transgenic mice exhibited an important reduction in LTB4 content accompanied by reduced inflammation, as evidenced by reduced levels of MCP-1 and TNF-α. Taken together, the results of their study indicate that an HFD increased ω-6 concentration and consequently LTB4 levels, which in turn induced inflammation and IR. Furthermore, PGE2, a lipid mediator derived from arachidonic acid, as well as LTB4, is increased in obesity (18). Indeed, a previous study has demonstrated that PGE2 worsens insulin resistance induced by interleukin 6 in hepatocytes (19). Despite this, we did not deeply address this point in this manuscript because it was focused on LTB4/LTB4R1. Thus, further studies are necessary to elucidate the role of each lipid mediator in the development of IR in the context of obesity.

Increased levels of free fatty acids are certainly one of the mechanisms involved in the proinflammatory state that link obesity to insulin resistance (20). Indeed, palmitate, a saturated fatty acid, can induce proinflammatory M1 macrophage polarization, which in turn promotes insulin resistance (21). Thus, palmitate has been extensively used to induce inflammation and insulin resistance (22, 23). In contrast, oleate, an unsaturated fatty acid, can improve insulin sensitivity (24, 25). In this context, a study performed by Pardo and colleagues (26) observed that preincubation with culture medium of RAW 264.7 macrophages treated with palmitate was able to decrease insulin-induced IR and Akt phosphorylation in hepatocytes, while such effect was not observed if macrophages were loaded with oleate. They also showed that preincubation with palmitate increased proinflammatory cytokines in macrophages, which in turn induced ER stress in hepatocytes, while these effects were not seen with oleate preincubation. In addition, the culture medium of macrophages pretreated with oleate exhibited lower levels of LTB4 compared to the culture medium incubated with palmitate. In contrast, when LTB4 was added to oleate in preincubation of macrophages, the protection of insulin signaling was missing in hepatocytes. Thus, this study has confirmed by *in vitro* studies ω-3 is able to attenuate LTB4 (26). In accordance, a recent study *in vivo* showed that HFD enriched with ω-3 (eicosapentaenoic acid) downregulated LTB4 levels and inflammation in visceral adipose tissue of mice (27). In summary, these data point out those strategies that decrease ω-6/ω-3 ratio are promising to lower LTB4 levels, thus preventing inflammation and IR in obesity.

## LTB4/LTB4R1 Axis Promotes Proinflammatory Phenotype of B Cells Which in Turn Orchestrate Overall Inflammation in Adipose Tissue in Obesity

Despite macrophages being the most abundant immune cells infiltrated into tissues, other immune cells such as leukocytes, lymphocytes, neutrophils, and eosinophils, are also present and may participate in the inflammatory process associated with obesity and IR. In this context, Nishimura and coworkers (28) demonstrated that CD8+ effector T cells are increased, while CD4+ helper and regulatory T cells decrease in adipose tissue in obesity. Furthermore, the activation of CD8+ T cells allows the recruitment of macrophages and their polarization toward M1, which evidences a role of adaptive immunity in the development of IR associated with obesity. In addition, another cell of the adaptive immune system that plays a role in obesity-induced IR is the B lymphocyte, since it is in adipose tissue in obesity and its genetic depletion reduces HFD-induced IR (29, 30).

However, the underling mechanisms related to B cell recruitment to adipose tissue were not uncovered until an article published in 2018 (3). In their elegant study, Ying W and colleagues by using knock out mice models and immune cell transplants, showed that the LTB4/LTB4R1 axis drives the attraction of B2 cells to adipose tissue during HFD feeding and directly stimulates a proinflammatory phenotype in these cells, promoting IR. They also demonstrated that B2 cells orchestrate IR by inducing Th1 response in lymphocytes and macrophage polarization towards M1. Despite this, the authors also concluded that macrophages can also induce IR by other mechanisms than this connected to B2 cells, since macrophage depletion achieved by using clodronate resulted in more pronounced effects in insulin sensitivity. Furthermore, their study also evidenced that the pivotal source of LTB4 are adipocytes because B cells still accumulate in the adipose tissue after depletion of macrophages and T cells. However, as adipose tissue also presents eosinophiles, neutrophiles and other immune cells (3, 31), we cannot rule out their participation, besides adipocytes, in LTB4 synthesis during obesity development. Also, as TNF-α, IL-6, and IL-1β increase in obesity by several mechanisms such as TLR4 activation, ER stress, among others (32–34), it is reasonable to assume that the increased levels of these cytokines can also enhance LTB4 synthesis and action. We also hypothesized that LTB4 and proinflammatory cytokines may work in a positive feedback loop. However, this point deserves its own review article. Taken together, these results unveiled that different immune cells orchestrate adipose tissue inflammation during obesity development, and shed light on LTB4R1 as a potential therapeutic target to improve IR.

## Conclusions

In conclusion, the studies of the Li and colleagues group discussed in this manuscript highlighted that several types of immune cells coordinate adipose tissue inflammation during obesity development and that LTB4/LTB4R1 has an important role in the inflammation-induced IR by a mechanism that involves JNK activation. Thus, further studies should investigate potential strategies to blunt LTB4-LTB4R1 (3, 11) (**Figure 1**). We also addressed articles regarding the relationship between PUFAs and LTB4 (17, 26). These data allow a conclusion that a high ω-6/ω-3 ratio increases LTB4 levels, i.e., collaborates with development of inflammation and IR in obesity (16), thus strategies that lower ω-6/ω-3 ratio are promising to reduce LTB4 levels and therefore deserve further investigation.

## Author Contributions

IC and AO contributed to discussion, and wrote, reviewed, edited manuscript. Both authors contributed to the article and approved the submitted version.

## Funding

This work was supported by grants from Fundação de Amparo à Pesquisa do Estado de São Paulo (FAPESP), Conselho Nacional de Pesquisa (CNPq) (Instituto Nacional de Ciência e Tecnologia - Obesidade e Diabetes) and was financed in part by the Coordenação de Aperfeiçoamento de Pessoal de Nível Superior - Brasil (CAPES) Finance Code 001.

## Conflict of Interest

The authors declare that the research was conducted in the absence of any commercial or financial relationships that could be construed as a potential conflict of interest.

## Publisher’s Note

All claims expressed in this article are solely those of the authors and do not necessarily represent those of their affiliated organizations, or those of the publisher, the editors and the reviewers. Any product that may be evaluated in this article, or claim that may be made by its manufacturer, is not guaranteed or endorsed by the publisher.
